# Effective Connectivity between Major Nodes of the Limbic System, Salience and Frontoparietal Networks Differentiates Schizophrenia and Mood Disorders from Healthy Controls

**DOI:** 10.3390/jpm11111110

**Published:** 2021-10-28

**Authors:** Sevdalina Kandilarova, Drozdstoy St. Stoyanov, Rositsa Paunova, Anna Todeva-Radneva, Katrin Aryutova, Michael Maes

**Affiliations:** 1 Department of Psychiatry and Medical Psychology and Research Institute, Medical University of Plovdiv, 4002 Plovdiv, Bulgaria; drozdstoy.stoyanov@mu-plovdiv.bg (D.S.S.); rositsa.paunova@mu-plovdiv.bg (R.P.); anna.todeva@mu-plovdiv.bg (A.T.-R.); katrin.aryutova@phd.mu-plovdiv.bg (K.A.); dr.michaelmaes@hotmail.com (M.M.); 2 Department of Psychiatry, Faculty of Medicine, Chulalongkorn University, Bangkok 10330, Thailand

**Keywords:** psychiatry, effective connectivity, depression, salience network, schizophrenia, mood disorders

## Abstract

This study was conducted to examine whether there are quantitative or qualitative differences in the connectome between psychiatric patients and healthy controls and to delineate the connectome features of major depressive disorder (MDD), schizophrenia (SCZ) and bipolar disorder (BD), as well as the severity of these disorders. Toward this end, we performed an effective connectivity analysis of resting state functional MRI data in these three patient groups and healthy controls. We used spectral Dynamic Causal Modeling (spDCM), and the derived connectome features were further subjected to machine learning. The results outlined a model of five connections, which discriminated patients from controls, comprising major nodes of the limbic system (amygdala (AMY), hippocampus (HPC) and anterior cingulate cortex (ACC)), the salience network (anterior insula (AI), and the frontoparietal and dorsal attention network (middle frontal gyrus (MFG), corresponding to the dorsolateral prefrontal cortex, and frontal eye field (FEF)). Notably, the alterations in the self-inhibitory connection of the anterior insula emerged as a feature of both mood disorders and SCZ. Moreover, four out of the five connectome features that discriminate mental illness from controls are features of mood disorders (both MDD and BD), namely the MFG→FEF, HPC→FEF, AI→AMY, and MFG→AMY connections, whereas one connection is a feature of SCZ, namely the AMY→SPL connectivity. A large part of the variance in the severity of depression (31.6%) and SCZ (40.6%) was explained by connectivity features. In conclusion, dysfunctions in the self-regulation of the salience network may underpin major mental disorders, while other key connectome features shape differences between mood disorders and SCZ, and can be used as potential imaging biomarkers.

## 1. Introduction

Mental illness can be defined as a complex construct integrating the continuity between a multifaceted phenomenological presentation and an incomplete theoretical knowledge on their morphological substrate and multifactorial etiopathophysiology [1]. There have been various conceptual attempts for systematization of psychiatric disorders, with two major approaches: a categorical one, which differentiates distinct nosological entities, such as schizophrenia (SCZ), bipolar disorder (BD) and major depressive disorder (MDD); and a dimensional approach, which defines mental illness as a continuum from adaptive to discordant behavioral patterns. Both formulations, however, are heretofore insufficiently validated by objective scientific findings. This deficiency can be explained both by the heterogeneity of research observations, and the diversity of the symptomatologic presentation, as well as the common comorbidity seen in psychiatric clinical practice [1]. 

Schizophrenia and bipolar disorder share many similarities and common features which support the continuum hypothesis. Both SCZ and BD demonstrate a high degree of genetic transmissibility supported by data from family and twin studies suggesting hereditary overlap, along with gene susceptibility markers located on the same chromosomes, and some similarities in neurotransmitter dysfunction [2]. Noto et al. (2019) reported that first-episode psychosis, which later evolves into schizophrenia and bipolar disorder, is characterized by a cytokine storm which is somewhat more pronounced in subjects who will later develop schizophrenia. The expanded continuum hypothesis was recently supported by the study of Sorella et al. [3]. They found clear evidence in SCZ and BD of a shared altered network of brain areas (including ventro-temporal and medial parieto-occipital areas, as well as portions of the cerebellum and the middle frontal gyrus), which could represent the neural underpinnings of an altered interpretation of reality connected with psychosis. This neural evidence, obtained by using magnetic resonance imaging (MRI), is supported by convergent neuropsychological evidence, obtained using cognitive tests, and is proposed to form a common “psychotic core” shared by SCZ and BD. Similarly, the authors report neural and psychological evidence for a “cognitive” core, and less so for an “affective” core. 

On the other hand, the scientific framework of mood disorders is shaped over two major theoretical concepts: the first separates MDD and BD into distinct categorical entities [4], and the other defines these classes as a dimensional continuum [5]. In addition, both disorders have complex and multifactorial etiopathophysiology (e.g., neurobiological, immunological, genetic, etc.) [6]. Contemporary psychiatric classifications group mood disorders into bipolar disorder with two distinct subtypes (BD type I and BD type II), and major depressive disorder. A recent study by Guo et al. demonstrated that, in terms of clinical symptoms, six major domains of Overactivation, Psychomotor Acceleration, Distraction/Impulsivity, Hopelessness, Retardation and Suicide Tendency can describe the three diagnostic groups in a continuum from low to high [7]. The existence of the so-called mixed states is an additional support for the continuity of the affective spectrum. In order to test the continuum hypothesis of mood disorders, Benazzi et al. explored the distribution of the intra-depression hypomanic symptoms between BD type II and MDD and failed to find the expected bi-modality which would support the categorical approach [8]. Their findings are mirrored by a similar non-bimodal distribution-of-lifetime manic/hypomanic symptoms in BD-I and MDD, and of the intra-mania depressive symptoms [9,10]. 

Nevertheless, nomothetic network analysis shows that a shared core (i.e., a statistically defined unidimensional factor) underpins unipolar and bipolar disorder, namely the interconnected reoccurrence of (hypo)manic and depressive episodes and suicidal behaviors [6]. This indicates that unipolar depression and bipolar disorder are the same illness and that staging of the illness contributes to different phenotypes [6]. This necessitates transdisciplinary methods for research, including the nomothetic networks approach as a bottom-up integration of horizontal and vertical levels of explanation [1,11]. 

However, heretofore, the results from numerous scientific inquiries regarding the connectome features of mood disorders and SCZ remain heterogeneous. For instance, Ambrosi et al. found a significant reduction of the resting-state functional connectivity (rsFC) between the left insula and the left mid-dorsolateral prefrontal cortex, as well as between bilateral insula and right frontopolar prefrontal cortex, in patients with bipolar depression as opposed to patients with unipolar depression and healthy individuals [12]. Moreover, in the same study, a decreased functional connectivity was established between the right amygdala and the left anterior hippocampus in participants with depression in the context of MDD compared to individuals with BD and healthy controls. However, Anand et al. observed similar alterations in unipolar and bipolar depression, namely a low rsFC between the pregenual anterior cingulate cortex and the dorsomedial thalamus in comparison with healthy controls [13]. 

In terms of large-scale networks, unipolar depression has been associated with increased functional connectivity in the Default Mode Network (DMN) and reduced rsFC between the cingulo-opercular network and DMN domains, whereas a higher rsFC in the frontoparietal network was observed in bipolar depression [14,15]. On the other hand, alterations in the static and dynamic functional connectivity strengths in the frontal–striatal–thalamic circuits (in BD) and within the DMN/sensorimotor network (in MDD) have also been demonstrated as possible differentiating biomarkers between these disorders [16].

SCZ and BD, on the other hand, demonstrate both shared and divergent characteristics of the connectome. For instance, the amygdala and prefrontal cortex appear to play important roles in both SCZ and BD. Liu et al. discovered that the rsFC between the amygdala and the dorsolateral PFC was significantly decreased in the SCZ group, whereas the rsFC between the amygdala and the ventrolateral PFC was significantly decreased in the BD group, suggesting that this dorsal vs. ventral PFC differentiation in amygdala–PFC connectivity might be used as a potential marker for differential diagnosis during the early stages of the diseases [17]. 

Moreover, Li et al. [18] found that both bipolar and schizophrenic patients had higher rsFC from the insula to the bilateral frontal pole and thalamus, the left middle frontal gyrus and the hippocampus when compared to the healthy controls. They also found that the bipolar group exhibited higher connectivity from the insula to the perigenual anterior cingulate cortex, whereas the schizophrenic group had higher connectivity from the insula to the right caudate and to the left middle frontal gyrus (an area that is situated on the lateral prefrontal cortex). These findings suggest that the insula plays a significant role in explaining the similar pathophysiology of bipolar disorder and schizophrenia that is supported by shared insular connectivity abnormalities patterns in both disorders. However, insular functional connectivity also has disorder-specific characteristics, which may point to potential pathways for differentiation during the early phases of the disease.

Despite the research outlined thus far, there is no definite answer to the question of whether the connectome differences between mentally healthy individuals and psychiatric patient groups are mainly quantitative or qualitative. The best way to explore this is by using a machine learning technique, namely Soft Independent Modeling by Class Analogy (SIMCA) [19,20]. SIMCA allows us to compute principal-component SIMCA models around the diagnostic classes based on connectome features and to compute the distance between the class models, whereby a large distance indicates qualitative differences between the classes [19,20,21]. 

Hence, this study was designed to examine whether there are quantitative or qualitative differences in the connectome between psychiatric patients and controls, on one hand, and to delineate the connectome features of MDD, SCZ and BD and the severity of the illness, on the other. To achieve our goals, we performed an effective connectivity analysis of resting-state functional MRI data of three groups of patients presenting with the abovementioned psychiatric diagnostic classes and a group of healthy controls. We have focused on effective connectivity, which delineates the influence that one neural system exerts over another, thereby reflecting a direct causal influence instead of functional connectivity, which discloses only the correlation between the BOLD signals derived from different brain regions [22]. In addition, we employed the spectral Dynamic Causal Modeling (spDCM) method [23], which estimates effective connectivity from the cross-spectra of the fluctuations in neuronal states rather than from their time courses directly, as is the case with stochastic DCM [24].

## 2. Subjects and Methods

### 2.1. Subjects

A hundred and one subjects were recruited for the present study, and they were divided into four groups: healthy controls, and patients with SCZ, BD or MDD. Each participant was assessed by experienced psychiatrists (D.S. and S.K.), using a general clinical interview and the structured Mini International Neuropsychiatric Interview (M.I.N.I. 6.0) [25]. In addition, the Montgomery–Åsberg Depression Rating Scale (MADRS) [26] and the Positive and Negative Syndrome Scale (PANSS) [27] were implemented in depressed and schizophrenic patients, respectively. The clinical diagnosis was established based on the interview, the available medical documentation and, in some cases, additional information from accompanying family members. The DSM-IV TR criteria were applied. Severity of illness was measured by using the Clinical Global Impression scales for severity (CGI). 

The SCZ group included subjects with a current psychotic episode, while patients with BD and MDD were suffering from a depressive episode at the time of recruitment. Psychiatric comorbidities, such as panic disorder, agoraphobia, social phobia, generalized anxiety disorder, obsessive–compulsive disorder, post-traumatic stress disorder, eating disorders (anorexia and bulimia) and alcohol or other substance-use disorders, as well as dissocial personality disorder identified with the M.I.N.I. interview, were excluded. 

The severity of depression was measured with the MADRS. For the current study, a cutoff value for the total score of 20 was used as an inclusion criterion for mood disorders. Severity of the psychotic symptoms was assessed with PANSS, providing detailed scoring of positive, negative and general symptoms. The psychotic symptoms ratings of P1 (delusions) and/or P6 (suspiciousness) had to exceed 3 to ensure the severity required. Nevertheless, in the current study, we computed an index of overall severity of schizophrenia as z (sum of P1 (delusions) + P2 (conceptual disorganization) + P3 (hallucinatory behavior) + P6 (grandiosity) + z (sum of N1 (blunted affect) + N2 (emotional withdrawal) + N3 (poor rapport) + N4 (passive social withdrawal) + N5 (difficulty in abstract thinking) + N6 (lack of spontaneity) + N7 (stereotyped thinking)). Both schizophrenic and depressed patients were taking stable doses of their antidepressant and/or antipsychotic medication for the preceding two weeks. 

Exclusion criteria were the following: age under 18 or above 65 years, presence of metal implants or body grafts (e.g., pacemaker) incompatible with MRI, history of a psychiatric disorder (for the healthy controls), comorbid psychiatric disorder as identified by the clinical interview and the M.I.N.I., severe somatic or neurological disease, and traumatic brain injury with loss of consciousness. Written informed consent complying with the Declaration of Helsinki was obtained from each participant prior to inclusion. The study protocol was granted approval by the University’s Research Ethics Committee (No. R-2172/03.04.3015).

### 2.2. Resting State MRI Acquisition and Analysis

A 3T MRI system (GE Discovery 750 w) was used for the scanning of the participants. A high-resolution structural scan was first obtained (Sag 3D T1 FSPGR, slice thickness 1 mm, matrix 256 × 256, relaxation time (TR)–7.2 ms, echo time (TE)–2.3 ms, flip angle 12°), followed by an eyes-closed resting-state functional scan (2D Echo Planar Imaging (EPI), slice thickness 3 mm, matrix 64 × 64, TR-2000 ms, TE–30 ms, 36 slices, flip angle 90°, 192 volumes).

The subsequent data analysis was performed with the SPM 12 software (Statistical Parametric Mapping, http://www.fil.ion.ucl.ac.uk/spm/, accessed on 4 March 2021), running on MATLAB R2020b for Windows. The preprocessing steps included realignment, co-registration with the structural scans, normalization to Montreal Neurological Institute (MNI) space and smoothing with a 6 mm full-width-at-half-maximum Gaussian kernel. 

Next, a general linear model (GLM) was applied to the time series, as well as the covariates of no interest: the six rigid-body-motion parameters, average white matter, and cerebrospinal-fluidsignal time series. The BOLD time series were then extracted for eight predefined right-sided regions of interest (ROI), using 6 mm radius spheres. These ROIs were the following (MNI coordinates given in brackets): ROI_1-anterior insula (AI) [38, 22, 3], ROI_2-inferior frontal gyrus (IFG) [50, 26, 16], ROI_3-middle frontal gyrus (MFG) [36, 42, 28] corresponding to dorsolateral prefrontal cortex (DLPFC), ROI_4-frontal eye field (FEF) [31, −5, 58], ROI_5-anterior cingulate cortex (ACC) [5, 45, 12], ROI_6-superior parietal lobe (SPL) [24, −54, 68], ROI_7-amygdala (AMY) [24, 3, −16], and ROI_8-hippocampus (HPC) [30, −11, −17]. 

Spectral dynamic causal modeling (spDCM) was performed with the abovementioned eight regions. We started with a fully connected model where each node was connected to each other node. The individual spDCM models were then jointly estimated using the Parametric Empirical Bayes framework, implemented in SPM12. In the last step the connectivity strengths (A-matrix) were extracted from the estimated spDCM models and further tested for statistical significance. The indexing of the connectivity values was as follows: A11 = self-inhibitory connection of the first ROI–anterior insula (AI⸧), A12 = influence of ROI_1 to ROI_2 (AI→IFG), etc. They can be excitatory (positive numbers) or inhibitory (negative numbers). 

### 2.3. Statistical Analysis

Difference between the study groups in scale variables were assessed by using the Kruskal–Wallis test or analysis of variance (ANOVA), followed by protected pairwise comparisons among treatment means. Associations between categorical variables were assessed by using analysis of contingency tables (χ^2^ test). We used multiple regression analysis to assess the significant (at *p* = 0.05) connectome data predicting the MADRS, CGI or OSOS scores, while allowing for the effects of age and sex. We used a combination of hierarchical (i.e., we defined the order to enter the predictor variables in the model based on the importance of the connectome data) and automatic multiple regression analyses, as explained when presenting the results of the regression analyses. The automated stepwise methods were performed with a p-to-entry of 0.05 and a p-to-remove of 0.06. All regressions were checked by using R^2^ change, as well as multicollinearity (using tolerance and variance inflation factor), homoscedasticity (the White and modified Breusch–Pagan tests) and multivariate normality (Cook’s distance and leverage). We employed stepwise binary logistic regression analysis with diagnostic classes as dependent variables and the connectome data as explanatory variables, while allowing for the effects of age and sex. We computed the odds ratio (OR) and corresponding 95% confidence intervals (CIs), as well as the Nagelkerke pseudo R^2^ value, which was used as an estimate of the effect size. Moreover, we bootstrapped all regression analyses (5.000 samples) and show the bootstrapped results if they would change the outcome of the model. The machine learning techniques, namely support vector machine (SVM), soft independent modeling of class analogy (SIMCA) and principal component analysis (PCA), followed by construction of a PC plot, were performed as explained previously [20]. Since the diagnostic classes were unbalanced, we employed a random oversampling approach with multiple copies of the smaller *n* class to achieve an equal split among the classes when conducting SVM and binary logistic regression analyses. All tests were two-tailed, and the significance was set at *p* = 0.05. The statistical analyses were performed by using IBM SPSS windows version 25, 2017 (ANOVA, χ^2^, regression analyses), and the Unscrambler X10.5.1 (PCA, PC plot, SVM, SIMCA).

## 3. Results

### 3.1. Sociodemographic Data

Table 1 shows the sociodemographic and clinical data of the patients and controls in this study. There were no significant differences in age, sex ratio and education between the four study groups. All three patient groups showed a higher CGI score than controls. The MADRS score was significantly higher in BD and MDD patients than in healthy controls. The OSOS score was significantly higher in SCZ patients than in controls. There were no significant differences in illness duration, episode duration, and number of episodes between the patient groups.

### 3.2. Connectome Features in Patients versus Controls

**Figure 1:**
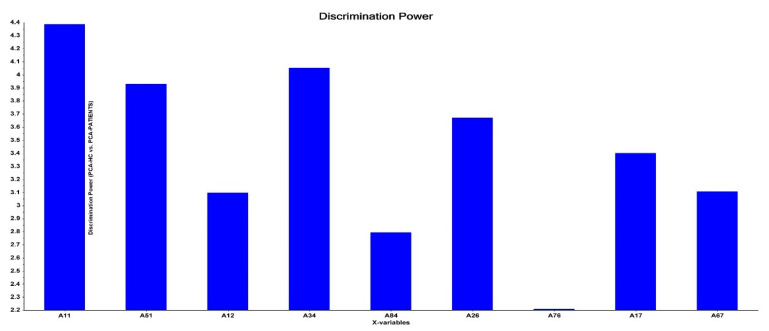
Discrimination power of the nine significant connections. (A11 = self-inhibition of the AI, A51 = ACC→AI, A12 = AI→IFG, A34 = MFG→FEF, A84 = HPC→FEF, A26 = IFG→SPL, A76 = AMY→SPL, A17 = AI→AMY, A67 = SPL→AMY.)

**Figure 2:**
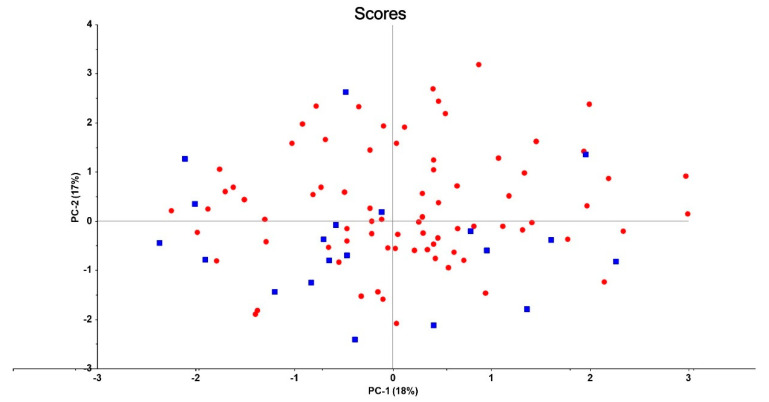
Plot of PC 1 and PC 2 explaining 35% of the variance.

A first SIMCA was performed by using all connectome data, and feature selection was performed based on the modeling, and discriminatory power of the connectome variables, resulting in nine variables with a significant modeling and discriminatory power. Consequently, we performed a second SIMCA which included only these 9 connectome features. Figure 1 shows that the top five discriminatory variables were (in descending order of discriminatory power) A11, A34, A51, A26 and A17. Nevertheless, SIMCA showed that the model-to-model distance was only 3.3, and that using those nine variables no significant classification ability could be achieved. Figure 2 shows the PC plot obtained by principal component analysis with the first two PCs explaining 35% of the variance. In this two-dimensional display of the multivariate dataset, no clear demarcation between patients and controls could be detected. Moreover, not one of the other combination of PCs showed any significant street between both groups. Using the same nine variables, we found that SVM showed a training accuracy of 96.4% and a validation accuracy of 87.1%. 

Consequently, we performed binary logistic regression analyses, which introduced all AI and ACC connectome data. After performing automatic regression analyses with feature selection, only two AI features were significant (A11 = self-inhibition of the AI and A17 = AI→AMY) and no ACC features. After this first step, we subsequently added the amygdala connectome features and performed another automatic regression, and found that A76 (AMY→SPL) and A37 (MFG→AMY) were additional significant features. Thereupon, we added the hippocampal feature set and found that A84 (HPC→FEF) was another significant connectome feature. After consequently adding the remaining feature sets while conducting feature selection, we delineated six significant predictor variables, namely A11 (AI self-inhibition), A34 (MFG→FEF), A84 (HPC→FEF), A76 (AMY→SPL), A17 (AI→AMY) and A37 (MFG→AMY) (see Table 2, Model #1). A11 and A76 were inversely associated with psychiatric disorders versus controls, and A34, A84, A17 and A37 were positively associated. By inference, SIMCA, SVM and binary logistic regression analyses share A11, A34, A84, A76 and A17 as discriminatory variables, while logistic regression revealed that A37 was another predictor variable. A51 (ACC→AI), A26 (IFG→SPL), and A67 (SPL→AMY) were significant discriminators, as detected by SIMCA/SVM, but not by binary regression. 

In order to examine the connectome predictors of both mood disorders (MOOD) versus controls, we entered the 5 variables delineated by the first regression in Table 2 together with A51, A26 and A67 in an automatic regression analysis and consequently added the other connectome feature sets in the same order as described above. In Table 2, Model #2 (MOOD vs. HC) shows that mood disorders were best predicted by five variables, namely A11, A34, A84, A17 and A37, with a pseudo R^2^ of 0.487 and an accuracy of 73.3% (sensitivity = 73.2%, and specificity = 73.3%). A34, A84, A17 and A37 were positively associated with mood disorders, whereas A11 was inversely associated. None of the other feature sets or connectome variables added important information.

To delineate the significant predictors of SCZ versus controls we followed the same procedure with the limit of maximal three explanatory variables. In Table 2, Model #3 (SCZ vs. HC) shows that two variables significantly discriminated SCZ from controls, namely A17 (inversely associated) and A76 (positively associated with a pseudo R^2^ of 0.316). None of the other connectome features had significant discriminatory power.

### 3.3. Connectome Features as Predictors of Severity of Illness

To delineate the connectome features that best predicted the severity of psychiatric illness (the CGI score), we performed automatic multiple regression analyses with the CGI score as dependent variable and the connectome data as explanatory variables, while allowing for the effects of age and sex (entered as a dummy variable). First, we entered all variables which were significant in the logistic regression analysis separating patients from controls. Table 3, Model #1 shows that only two of those variables significantly predicted CGI, namely A17 and A84, which together explained 14.9% of the variance in the CGI score. Sex and age were not significant in this regression analysis. Consequently, we first included the ACC data, and then we added the amygdala connectome data, performed automatic regression analysis, and found that two more features were significant predictors, namely A56 and A65. Consequently, adding the other feature sets showed that none of the remaining features was significant. In Table 3, Model #2 shows that 29.8% of the variance in the CGI score was explained by the regression on A17, A84, A56 and A65 (all positively correlated).

**Figure 3:**
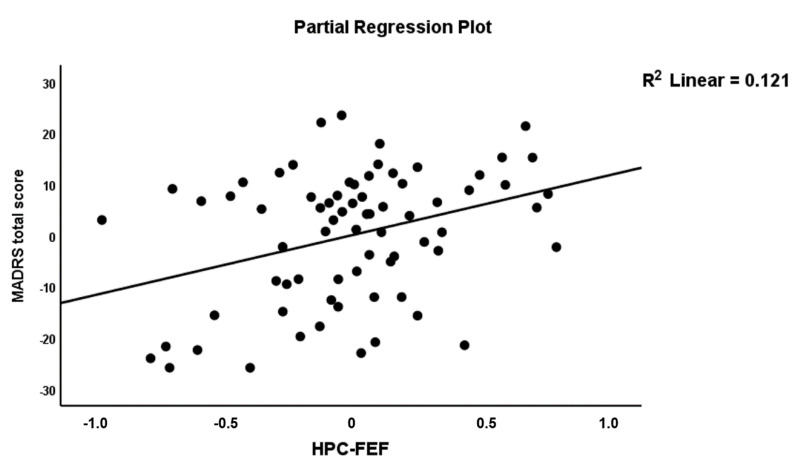
Partial regression plot of the MADRS score on the HPC→FEF connection.

**Figure 4:**
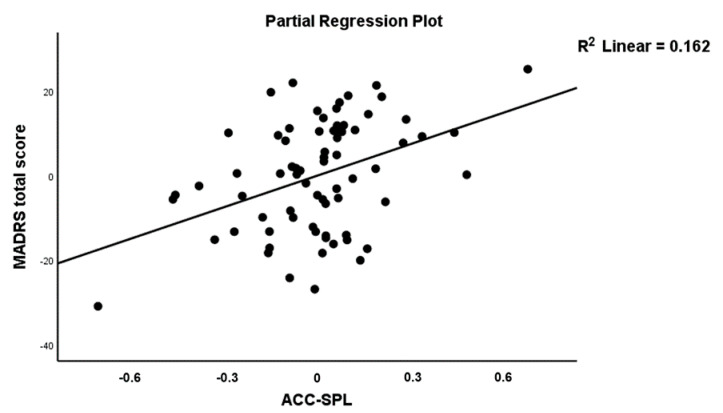
Partial regression plot of the MADRS score on the ACC→SPL connection.

To define the best connectome predictors of the MADRS, we performed the same procedure as explained above (see CGI score). These automatic regression analyses showed that five connectome features were incorporated in the final model (see Table 3, Model #3) and explained 31.6% of the variance in the MADRS data. A84, A56, A65 and A67 were positively associated with the MADRS, and A11 was inversely associated with the MADRS score. Figure 3 shows the partial regression of the MADRS score on A84 (HPC→FEF). Figure 4 shows the partial regression of the MDRS score on A56 (ACC→SPL).

**Figure 5:**
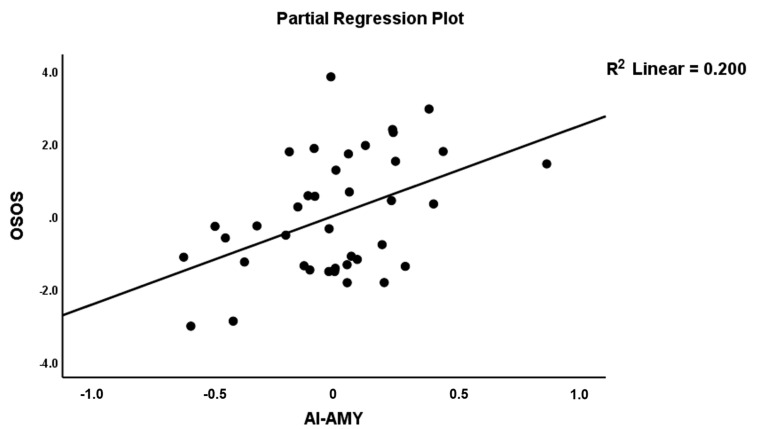
Partial regression plot of the OSOS score on the AI→AMY connection.

**Figure 6:**
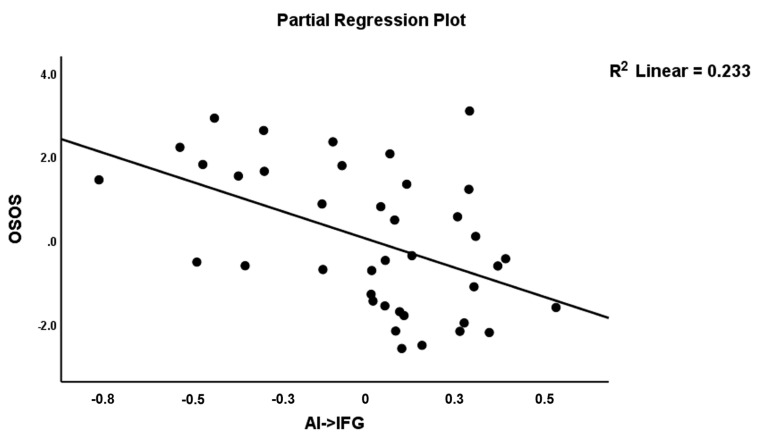
Partial regression plot of the OSOS score on the AI→IFG connection.

To examine the connectome features of OSOS, we first entered the two connectome variables that significantly discriminated SCZ from controls and found that A17 (positively) and A76 (inversely) were significantly associated with OSOS explaining 26.9% of its variance. Entering the other connectome feature sets revealed that A76 was no longer significant after considering the effects of two other variables, namely A12 and A26, which were both inversely associated with OSOS. As such, three connectome features explained 40.6% of the variance in OSOS, namely A17 (positively), A12 and A26 (both inversely). Figure 5 shows the partial regression of OSOS on A17 (AI→AMY), and Figure 6 shows the partial regression of OSOS on A12 (AI→IFG).

### 3.4. Connectome Features Discriminating Patient Subgroups

In Table 4, Regression #1 shows the results of a binary logistic regression analysis separating mood disorders from SCZ. To construct this final model, we first entered the amygdala, hippocampus and MFG feature sets and performed an automatic regression analysis resulting in three significant explanatory variables, namely A27, A23 and A76. Following this first step, we consequently added the superior frontal gyrus feature set, but no additional features were significant. Next, we added the IFG feature set and found that three additional IFG features could be added as discriminatory variables. The addition of the other feature sets did not reveal any other significant features. In Table 4, Regression #1 shows the final model, i.e., six connectome features significantly discriminated both groups with a pseudo R^2^ value of 0.604 and an accuracy of 83.1% (sensitivity = 75.0%, and specificity = 89.7%); A23 and A76 were positively associated and A21, A52, A25 and A27, inversely associated with mood disorders versus SCZ. Lastly, we performed a logistic regression analysis with MDD as a dependent variable and BD (no MDD) as a reference group and firstly entered the AI and ACC datasets. The final model (Table 4, Regression #2) shows that three connectome features were associated with MDD, namely A31 (positively), A26 and A57 (both inversely), with a pseudo R^2^ value of 0.547 and accuracy of 80.4%. 

## 4. Discussion

The first major finding of our study points to highly significant connectome differences between patients and controls, as demonstrated by using SIMCA and SVM. Nevertheless, these differences were more quantitative than qualitative because the distance between both SIMCA models constructed around the SCZ and control classes was not that large [20,21]. These findings contribute to the growing evidence of quantitative changes along a spectrum from health to mental illness. These advanced machine learning techniques were able to define a model consisting of nine connectome features, reaching a training accuracy of 96.4% and a validation accuracy of 87.1%, as demonstrated by SVM. The top five features of the model included the self-inhibition of the AI (A11), the MFG→FEF (A34), the ACC→AI (A51), the IFG→SPL (A26) and the AI→AMY connections (A17). The binary logistic regression analysis, on the other hand, identified the AI self-inhibition (A11), the MFG→FEF (A34), the HPC→FEF (A84), the AMY→SPL (A76), the AI→AMY (A17) and the MFG→AMY (A37) connections as significant discriminators between patients and healthy individuals. Notably, three of the features were detected in both SIMCA and binary logistic regression, namely the self-inhibition of the AI, the MFG→FEF and the AI→AMY connectivity, and should be regarded as “authorities” or key connectome features. 

The role of the AI (part of the salience network), the AMY (major node of the limbic system), the MFG (DLPFC) and the FEF (the central executive network) in the development of various psychiatric disorders has been suggested by numerous studies; however, not all interactions have been completely delineated. For instance, the AI as part of the SN regulates the dynamic switch between the DMN and the central executive network and is essential for the rapid change of focus between internal and external stimuli. By integrating sensory, emotional and cognitively charged information, the SN engages in complex processes, such as communication, social behavior and self-awareness [28]. In schizophrenia and high-risk individuals for psychosis, there is impaired functional connectivity (FC) in the nodes of the SN, as well as aberrant interactions of the SN with other large brain networks [29,30]. Depression, on the other hand, has been associated with decreased FC within the SN, and the severity of symptoms correlated with decreased intrinsic FC of the right AI [31]. Moreover, decreased functional connectivity between DLPFC and insula was found in subjects with subthreshold depression compared to healthy controls [32]. In a previous study, we have found decreased effective connectivity between the AI and the MFG as well as an aberrant connection (non-existent in healthy individuals) from the AMY to the AI in a sample of unipolar and bipolar depressed patients [33]. 

In short, it seems that the dysfunction of the SN is in line with the clinical presentation and the suggested hypothesis of symptom formation in both mood and psychotic disorders—with the predominance of externalizing mental representations in SCZ leading to paranoid symptoms and overrepresented internalizing in depression that leads to self-defeating depressive symptoms. This might explain why the self-inhibitory properties of the AI in our study were identified as the major connectome feature contributing to the distinction between healthy individuals and psychiatric patients presenting with psychotic or depressive symptoms. 

The other significant connection in our results involved the influence of the DLPFC on the FEF, two regions considered to be part of the central executive network (known as well as the frontoparietal network) and the dorsal attention network or dorsal frontoparietal network, respectively. Several lines of research support the role of the DLPFC in the pathophysiology of both SCZ and depression. DLPFC dysfunction was found to characterize SCZ patients during a high cognitive control task, along with significant impairments in the functional connectivity between the dorsolateral prefrontal cortex and other task-relevant brain regions. In addition, cognitive performance, behavioral disorganization and global functioning demonstrated significant correlations with DLPFC functional connectivity [34]. Moreover, a lower ratio of N-acetyl aspartate to creatine in the left DLPFC was associated with the cognitive deficits in patients with first-episode SCZ, and was suggested to be an early biochemical marker for the cognitive impairment in schizophrenia [35]. 

Apart from the classical role of the DLPFC in “cognitive” or “executive” functions, such as working memory, intention formation, goal-directed action, abstract reasoning and attentional control [36], which are often impaired in SCZ, there is an increasing understanding of its involvement in the regulation of emotions, as well [37], and more specifically of the valence of emotional experiences [38]. Additionally, the DLPFC is responsible for suppression of posterior cingulate cortex overactivation, which is considered to underlie depressive ruminations [39]. Decreases of gray-matter volumes, along with disruptions of both DLPFC activity during task performance and functional connectivity during rest have been demonstrated in depression [40,41,42]. Moreover, the region is used as a target for different treatment techniques, such as neuro-feedback [43], and transcranial magnetic stimulation alleviating the symptoms of depression [44]. 

The third significant feature of the model distinguishing between healthy controls and mentally ill patients was the connection arising from the AI to the AMY. The role of the AI as part of the SN that has been implicated in the detection and integration of emotional and sensory stimuli, and its contribution to the pathophysiology of the diagnostic entities under study has been underlined repeatedly throughout the discussion hitherto. Therefore, we will focus our further attention on the amygdala as a key node of the limbic system, along with the hippocampus, hypothalamus, OFC and ACC [45]. A variety of structural, functional and connectivity abnormalities of the AMY have been demonstrated in SCZ, bipolar and unipolar depression [46,47,48,49]. Recent meta-analysis reported consistent findings of reduced left, right and total amygdala volumes in SCZ relative to both healthy controls and bipolar subjects, while such abnormalities were not confirmed in bipolar patients. Studies of the uncinate fasciculus tract (which connects the AMY with the medial- and orbitofrontal cortices) showed comparable degrees of reduced fractional anisotropy in both SCZ and bipolar patients. In addition, decreased amygdala-orbitofrontal cortex functional connectivity was generally a characteristic of SCZ, while in bipolar disorder the findings were inconsistent [50]. 

A review of emotion processing fMRI studies in major depression has confirmed the often-reported increased amygdala activation to negative and arousing stimuli which typically normalizes with antidepressant treatments [51]. Comparison studies suggested greater activation of the amygdala toward negative emotional stimuli in MDD compared to BD, with the opposite pattern during exposure to positive emotional stimuli of diverse types (facial expressions, even in subliminal presentations, and autobiographical memories) [15]. Lower resting state FC (rsFC) between the right amygdala and the left anterior hippocampus was observed in MDD compared to BD and controls [12]. BD patients showed increased rsFC between left amygdala and left anterior supramarginal gyrus when compared to healthy controls and MDD [52]. Overall, the literature is consistent in stating that amygdala dysfunction plays a crucial role in the pathophysiology of both unipolar and bipolar depression; however, there is less support for the specific alterations distinguishing both classes. 

In conclusion, our findings suggest that there are three major connectivity features distinguishing mentally healthy individuals from psychiatric patients: first, the self-regulatory properties of the AI (securing the balance between the internally and externally focused attention); second, the communication between the DLPFC and the FEF (providing appropriate executive functioning and attention); and third, the influence that the AI exerts on the AMY (the salience network regulation of the emotion processing). Our results are supported by numerous findings of structural and functional disruptions of the abovementioned brain regions in all three diagnostic classes under study. Moreover, studies on the neural substrates of general psychopathology outline a role for delayed maturation of limbic and default mode connectivity and more generally reduced between-network connectivity, leading to a compromised ability to integrate and switch between internally (somatosensory–motor networks, DMN) and externally (executive networks) focused tasks [53].

In addition, the overall severity of illness, as measured with CGI, was predicted by connectome features, including, again, the anterior insula and amygdala nodes, as well as the hippocampus, the frontal eye field, and the bidirectional connection between the superior parietal lobe and the anterior cingulate cortex. As mentioned earlier, the ACC is a major node of the limbic network as well as the SN, and both structural and functional anomalies of this brain area have been reported in major psychiatric disorders, more so in bipolar and unipolar depression but in SCZ, as well [15,54,55,56,57]. A recent meta-analysis by Goodkind et al. [58] compared structural imaging studies across major psychiatric disorders, and demonstrated a shared pattern of reduced grey matter volume in dorsal anterior cingulate cortex and bilateral anterior insula. The only significant difference across diagnostic classes was the more pronounced hippocampus/amygdala gray matter loss in MDD compared to BD, anxiety and obsessive–compulsive disorder.

The second major finding in our study was that SCZ could be discriminated from healthy controls, using the AI→AMY (A17) and the AMY→SPL connections (A76). In addition, the OSOS was predicted by the connections from the AI to both the AMY (A17), and the IFG (A12), and from the IFG to the SPL (A26). As discussed earlier, the structural, functional and connectivity findings point to the involvement of both the anterior insula and the amygdala in the pathophysiology of SCZ. In addition, a recent study using combined voxel-based morphometry and resting-state functional connectivity reported that early stage SCZ patients demonstrated a significantly decreased gray matter volume in both bilateral AI and ACC compared to the HC group. Furthermore, a marked reduction of the functional connectivity within the SN was found in the SCZ group. These convergent morphological and functional deficits in the SN were significantly associated with hallucinations [56]. In line with our findings, a most recent resting state fMRI study reported decreased FC of the right amygdala with the anterior insula in both high-risk subjects and first episode schizophrenia [49]. Moreover, the positive symptom scores of the PANSS scale were correlated with the FC within the right AI during the state of psychosis [29].

We also found that the severity of SCZ was associated with changed connectivity in the SPL and the IFG nodes. The superior parietal lobe plays a key role in different brain functions, including visuomotor, cognitive, sensory, higher order, working memory, attention and visuospatial perception, including the representation and manipulation of objects [59,60,61]. The inferior frontal gyrus is functionally a part of the ventrolateral prefrontal cortex (VLPFC), which is involved in cognitive control and motor-response inhibition [62], as well as in emotion regulation, and its activation correlates with the intensity of the emotional stimuli [63]. Moreover, the activation of the right VLPFC seems to be crucial for the successful implementation of emotion-regulation strategies, such as affect labeling or cognitive reappraisal, which can eventually reduce the activation of the amygdala to negative stimuli [64,65]. Compared to healthy controls both SCZ patients and their unaffected siblings demonstrated hypoactivation in VLPFC, insula and middle temporal gyrus when reappraising negative pictures [66]. Similarly, reduced activation of the VLPFC was found during conscious downregulation of negative emotions in schizophrenic compared to bipolar patients [67]. Meta-analytic data show that, when emotional facial stimuli were contrasted to neutral stimuli, SCZ patients displayed hypoactivation throughout the entire facial-affect-processing network and increased activation in visual-processing regions within the cuneus [47]. Thus, we may suggest that the severity of schizophrenia symptoms in our study was associated with the connectivity between regions implicated mainly in emotion regulation. 

The third major finding of the current study was that five connectome features could discriminate healthy controls from mood-disorder patients, namely the self-inhibition of the AI (A11), the connections from the MFG and the HPC to the FEF (A34 and A84), and the influences exerted upon the AMY by both AI (A17) and MFG (A37). The role of the structural and functional abnormalities of the amygdala and the anterior insula in the pathophysiology of depression was discussed earlier (see above). The influences exerted by the MFG (DLPFC) and HPC onto the FEF reflect the top-down and bottom-up regulation of attention by the cognitive and affective systems, respectively. As depression encompasses both cognitive and affective symptoms, our findings further support the research so far outlining the impairment of both the cognitive control (for example, hypoactivation of the DLPFC during task performance [40,68] and the emotional response, e.g., the increased activation of the limbic system in response to negative stimuli [51]). 

The interactions of the SN (AI) and the central executive network (MFG) with the limbic system (AMY) seem to represent a key feature of mood disorders. A possible clinical correlate of the dysfunctional communication between those three major networks might be the well-known negative bias in depression [69,70]. The basis of it is suggested to be the failure of the DLPFC to exert appropriate top-down regulation of the AMY response, along with the bottom-up emotional expression dysfunction characterizing depression [71]. In accordance with this explanation are the reports of reduced functional and effective connectivity in both MDD and BD [72,73]. 

An additional finding of our study consists of the MADRS score being predicted by the following connections: HPC→FEF (A84), ACC→SPL (A56), SPL→ACC (A65) and SPL→AMY (A67), which were positively associated with the score, and self-inhibition of the AI (A11), which was inversely associated. The main nodes involved in these significant connections include key limbic regions, such as the HPC, ACC and AMY, as well as the SPL, which is part of the association cortex. Previous studies exploring the neural correlates of symptom severity of depression have found various positive associations: (1) decreased intrinsic FC within the right AI [31], (2) increased FC of the DMN [74], (3) increased amplitude of low-frequency fluctuations (ALFF) values of the left dorsal medial PFC [75], (4) ALFF values of the right superior frontal gyrus [76] (while the same study found a negative correlation with the ALFF values of the left insula), (4) increased FC of bilateral dorsal medial PFC [77] and (5) reduced perfusion in the DMN (the posterior cingulate cortex and the right inferior parietal lobe) [78]. On the other hand, our finding of negative associations between AI self-regulation and the MADRS score is in line with the report of Manoliu et al., who found that the decreased intrinsic FC within the right AI was positively associated with severity of symptoms [31]. 

Finally, in our study there were three connectome features significantly discriminating between MDD and BD, namely the IFG→SPL, the ACC→AMY and the MFG→AI connections. Once again, we detected the major nodes of the limbic system, namely the anterior cingulate cortex and the amygdala (emotion processing), along with the dorsolateral and ventrolateral prefrontal cortices (cognitive control) and the balancing SN in the face of the anterior insula. Differentiating between unipolar and bipolar depression is a major unsolved clinical challenge in psychiatry, and it is not surprising that there is an abundance of studies comparing the two diagnostic entities in the search for potential structural, functional or connectivity markers, as was reported in a recent review by Han et al. [15]. Overall, the most convergent findings involve regions such as the amygdala, the anterior cingulate cortex and the prefrontal cortex, mainly DLPFC. Nevertheless, to the best of our knowledge, there are no other effective connectivity studies comparing unipolar and bipolar depression that employ spectral DCM by the time of the writing of the present text. 

However, in line with our findings is the reported association of bipolar depression with decreased FC between the insula and the DLPFC when compared to MDD and controls [12]. Our results can be viewed as complementary in the sense that they demonstrate the directionality of the differential connection, namely from the DLPFC to the AI. Moreover, in a recent study exploring the brain networks’ connectivity by means of group independent component analysis and graph theory, BD was associated with stronger FC and more efficient topological properties in the DLPFC, VLPFC and ACC when compared to MDD [79].

Earlier reports on resting state fMRI demonstrated that BD was associated with decreased ALFF in the left posterior insula and superior parietal lobule and increased amplitude of low-frequency fluctuations in the right dorsal anterior insula compared to MDD [80]. Another study revealed that individuals with BD showed lower fractional ALFF in the left medial and middle frontal gyrus compared to those with MDD [81]. Thus, along with previous findings, our results suggest that abnormalities in resting-state neural connectivity of the anterior insula, amygdala and PFC may be a useful marker for differentiating the depressive states of MDD and BD. 

In the final lines of this report, we want to point the reader’s attention to the fact that four out of the five connectome features that discriminate controls from mental illness are features of mood disorders, namely the MFG→FEF, the HPC→FEF, the AI→AMY and the MFG→AMY connections (A34, 84, 17 and 37). As can be easily seen, these involve major nodes of the SN, frontoparietal network, dorsal attention network and the limbic system. Only one of the connections was pertinent to SCZ, the AMY→SPL connectivity (A76), which reflects the influence of the limbic system on the association cortex, while the self-inhibitory connection of the AI (A11) is a feature of both mood disorders and SCZ. Thus, the shared A11 connectome feature supports the continuous theory, i.e., the self-regulation of the SN underpins mental illness, while the other features support qualitative differences between mood disorders and SCZ, and can be used as potential imaging biomarkers. 

Several limitations of the current study should be acknowledged. First, the sample size might not be sufficient to detect more subtle changes in connectivity. Second, since the medication status may have influenced the results, future studies on unmedicated patients are needed to establish the replicability of our findings. In addition, study samples should be enlarged to examine this issue, using machine learning and network analysis. It is worth noting that other sophisticated machine learning algorithms, such as Tensorflow, which incorporates a pre-training stage by using known control/patient data and non-linear regression or pattern recognition, may further improve the observed intensity of connections. 

## Figures and Tables

**Table 1:** Sociodemographic data.

	HC ^A^ (*n* = 21)	SCZ ^B^ (*n* = 24)	BD ^C^ (*n* = 23)	MDD ^D^ (*n* = 33)	KWT/F/χ^2^	df	*p*
Age–years (SD)	39.0 (13.1)	38.8 (14.0)	42.8 (11.9)	46.6 (13.9)	2.21	3/97	0.092 ^a^
Sex (M/F)	5/16	12/12	8/15	12/21	3.37	3	0.338 ^b^
Education-years (SD)	14.3 (2.0)	12.8 (2.4)	13.6 (2.3)	14.0 (2.3)	1.86	3/96	0.141 ^a^
CGI-S mean (SD)	1.0 (0.0) ^B,C,D^	4.29 (0.69) ^A^	4.56 (0.73) ^A^	4.39 (0.70) ^A^	KWT		<0.001 ^a^
MADRS mean (SD)	0.5 (1.3) ^C,D^	-	30.3 (6.1)	29.3 (7.0)	201.41	2/69	<0.001 ^a^
OSOS (z score)	−1.79 (0.) ^B^	1.82 (0.91) ^A^	-	-	KWT		<0.001 ^c^
Illness duration (months)	-	156.6 (116.1)	133.7 (91.8)	118.0 (93.7)	0.96	2/73	0.387 ^a^
Episode duration (weeks)	-	16.1 (16.7)	17.0 (18.5)	14.7 (16.6)	0.11	2/67	0.900 ^a^
Number of episodes	-	5.0 (4.5)	4.9 (4.6)	3.9 (4.0)	0.48	2/66	0.619 ^a^

SD, standard deviation; ^a^ one-way ANOVA; ^b^ χ^2^-test; ^c^ Two sample *t*-test, KWT, Kruskal–Wallis test; ^A,B,C,D^, pairwise comparisons between group means; CGI-S, Clinical Global Impression—Severity; MADRS, Montgomery–Åsberg Depression Rating Scale; OSOS, overall severity of schizophrenia.

**Table 2:** Results of linear logistic regression analysis with the psychiatric patients’ groups (SCZ, BD and MDD) as dependent variable, and controls as reference group.

Explanatory Variables	Nagelkerke Pseudo R^2^	χ^2^ (df) *p*-Values	B	Standard Error	Wald df = 1	*p*	Odds Ratio	95% CI
All patients vs. HC	0.448	56.83 (6)						
AI⸧ (A11)			−1.113	0.308	14.24	<0.001	0.31	0.17–0.57
MFG→FEF (A34)			0.722	0.252	8.23	0.004	2.05	1.26–3.37
HPC→FEF (A84)			0.684	0.231	8.77	0.003	1.98	1.26–3.12
AMY→SPL (A76)			−0.780	0.263	8.81	0.003	0.46	0.27–0.77
AI→AMY (A17)			1.261	0.282	20.02	<0.001	3.53	2.03–6.13
MFG→AMY (A37)			0.827	0.279	8.78	0.003	2.29	1.32–3.95
MOOD vs. HC	0.487	45.69 (5) <0.001						
AI (A11)			−1.348	0.401	11.27	0.001	0.26	0.12–0.57
MFG→FEF (A34)			1.105	0.350	9.97	0.002	3.02	1.52–6.00
HPC→FEF (A84)			0.950	0.295	10.40	0.001	2.59	1.45–4.61
AI→AMY (A17)			1.380	0.381	13.14	<0.001	3.97	1.89–8.38
MFG→AMY (A37)			1.180	0.371	10.11	0.001	3.26	1.57–6.74
SCZ vs. HC	0.316	12.16 (2) 0.002						
AI→AMY (A17)			0.802	0.382	4.41	0.036	2.23	1.06–4.71
AMY→SPL (A76)			−1.112	0.479	5.40	0.020	0.03	0.13–0.84

**Table 3:** Results of multiple regression analysis with the CGI, MDRS and OSOS as dependent variables and connectivity strengths as explanatory variables.

Dependent Variables	Explanatory Variables	B	A	*p*	F	df	*p*	R^2^
CGI	Model #1				6.03	2/69	0.004	0.149
	HPC→FEF (A84)	0.265	2.38	0.020				
	AI→AMY (A17)	0.279	2.51	0.014				
CGI	Model #2				7.10	4/67	<0.001	0.298
	AI→AMY (A17)	0.278	2.79	0.007				
	HPC→FEF (A84)	0.317	3.06	0.003				
	ACC→SPL (A56)	0.468	3.75	<0.001				
	SPL→ACC (A65)	0.299	2.42	0.018				
MADRS	Model #3				6.11	5/66	<0.001	0.316
	HPC→FEF (A84)	0.308	3.02	0.004				
	ACC→SPL (A56)	0.422	3.57	0.001				
	AI⸧ (A11)	−0.223	−2.17	0.034				
	SPL→ ACC (A65)	0.317	2.61	0.011				
	SPL→ AMY (A67)	0.223	2.11	0.038				
OSOS	Model #4				6.62	2/36	0.004	0.269
	AI→AMY (A17)	0.410	2.88	0.007				
	AMY→SPL (A76)	−0.332	−2.33	0.026				
OSOS	Model #5				7.96	3/35	<0.001	0.406
	AI→AMY (A17)	0.386	2.96	0.006				
	AI→IFG (A12)	−0.432	−3.26	0.002				
	IFG→SPL (A26)	−0.334	−2.52	0.016				

**Table 4:** Results of binary logistic regression analyses that delineate the connectome features of patient subgroups.

Explanatory Variables	Nagelkerke Pseudo R^2^	χ^2^ (df) *p*-Values	B	Standard Error	Wald df = 1	*p*	OR	95% CI
MOOD vs. SCZ	0.604	74.55 (6) <0.001						
IFG→AI (A21)			−6.272	1.625	14.89	<0.001	0.00	0.00–0.05
ACC→IFG (A52)			−3.935	1.239	10.08	0.001	0.02	0.00–0.22
IFG→ACC (A25)			−3.314	1.057	9.84	0.002	0.04	0.00–0.29
IFG→AMY (A27)			−4.430	1.422	9.71	0.002	0.01	0.00–0.19
AI→AMY (A23)			1.495	0.368	16.51	<0.001	4.46	2.17–9.17
AMY→SPL (A76)			1.687	0.470	12.90	<0.001	5.40	2.15–13.57
MDD vs. BD	0.547	29.16 (3) <0.001						
IFG→SPL (A26)			−5.39	2.632	9.76	0.002	0.01	0.00–0.13
ACC→AMY (A57)			−5.85	2.908	5.18	0.023	0.00	0.00–0.44
MFG→AI (A31)			1.36	0.675	8.10	0.004	3.90	1.53–9.96

## Data Availability

The dataset generated during and/or analyzed during the current study will be available from DAS upon reasonable request and once the dataset has been fully exploited by the authors.

## Abbreviations

ACC	Anterior cingulate cortex
AI	Anterior insula
AMY	Amygdala
BD	Bipolar disorder
CGI-S	Clinical global Impression—Severity scale
DCM	Dynamic Causal Modeling
DLPFC	Dorsolateral prefrontal cortex
DMN	Default mode network
FC	Functional connectivity
FEF	Frontal eye field
HC	Healthy controls
HPC	Hippocampus
IFG	Inferior frontal gyrus
MARDS	Montgomery–Åsberg Depression Rating Scale
MDD	Major depressive disorder
MFG	Middle frontal gyrus
OFC	Orbitofrontal cortex
OSOS	Overall severity of schizophrenia
PANSS	Positive and negative syndrome scale
PC/PCA	Principal component/principal component analysis
PFC	Prefrontal cortex
SCZ	Schizophrenia
SIMCA	Soft Independent Modeling by Class Analogy
SN	Salience network
SPL	Superior parietal lobule
SVM	Support vector machine
VLPFC	Ventrolateral prefrontal cortex
